# Development of Functional Soybean‐Enriched Cheese: Identification of Novel Angiotensin‐Converting Enzyme‐Inhibitory and Predicted Dipeptidyl Peptidase IV‐Related Peptides

**DOI:** 10.1002/fsn3.72155

**Published:** 2026-07-24

**Authors:** Mehtap Er Kemal, Mehmet Kemal, Hasan Temiz

**Affiliations:** ^1^ Department of Food Processing, Maçka Vocational School Karadeniz Technical University Trabzon Türkiye; ^2^ Department of Nutrition and Dietetics, Faculty of Health Science Karadeniz Technical University Trabzon Türkiye; ^3^ Department of Food Engineering, Faculty of Engineering Ondokuz Mayıs University Samsun Türkiye

**Keywords:** angiotensin‐converting enzyme inhibition, bioactive peptides, dipeptidyl peptidase IV, hybrid cheese, molecular docking, soybean beverage

## Abstract

This study investigated the peptidomic profiles and bioactivity potential of hybrid cheeses fortified with soybean beverage (0%, 13%, 26%, and 39%) and co‐fermented with *Lactobacillus helveticus* or *Lactobacillus acidophilus* over a 45‐day ripening period. Peptidomic profiling identified 102 peptide sequences, comprising 74 milk‐derived and 28 soybean‐derived peptides. Several soybean‐derived peptides, including DQMPRRF, NANSIIYAL, and QSKPNTIL, were identified for the first time in a cheese matrix. The highest angiotensin‐converting enzyme‐inhibitory (ACE‐I) activity was observed in the 39% soybean beverage cheese fermented with *L. helveticus* , suggesting that this strain promoted the release of bioactive peptide sequences from the hybrid dairy‐soy protein matrix. In silico docking analyses further indicated that selected soybean‐derived peptides, together with casein‐derived sequences such as FFVAPFPEVFGK, may interact favorably with angiotensin‐converting enzyme (ACE) and dipeptidyl peptidase IV (DPP‐IV) binding regions. These predicted interactions provide supportive structural evidence for the functional potential of the identified peptides, while not serving as direct confirmation of biological activity. Overall, this study highlights the potential of combining plant and dairy proteins with proteolytic starter cultures as a sustainable strategy for developing value‐added hybrid cheeses with potential relevance to dietary approaches targeting hypertension and type 2 diabetes.

## Graphical abstract

This graphical abstract summarizes the development of soybean‐enriched hybrid cheese, its fermentation with proteolytic starter cultures, peptidomic profiling by LC–MS/MS, and the identification of milk‐ and soybean‐derived peptides with angiotensin‐converting enzyme‐inhibitory activity and predicted dipeptidyl peptidase IV‐related bioactivity.

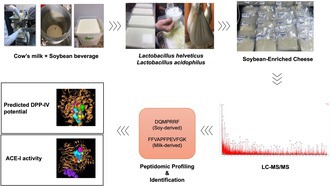

## 1 Introduction

Hypertension is a global health problem and primary risk factor for cardiovascular diseases, including coronary heart disease, peripheral arterial disease, and stroke. The incidence of hypertension is increasing due to factors such as population growth and aging. According to the World Health Organization, hypertension is estimated to affect approximately 1.28 billion adults worldwide (Fyfe‐Johnson et al. 2016). Angiotensin‐converting enzyme (ACE) converts angiotensin‐I to angiotensin‐II. This enzyme is key to the regulation of blood pressure, leading to vasoconstriction and increased blood pressure. In addition, ACE deactivates bradykinin, contributing to elevated blood pressure levels (Abedin et al. 2022). Consequently, the inhibition of ACE is a widely established and essential therapeutic mechanism for lowering blood pressure and mitigating cardiovascular risks.

Food‐derived peptides exhibit various bioactivities such as the mitigation of cardiovascular disease, cancer risk, and the reduction of diabetes incidence. Additionally, these peptides play a crucial role in inhibiting ACE, which is essential for the regulation of blood pressure (Olalere et al. 2023). Pharmaceuticals containing potent active ingredients like enalapril and captopril can be effective in blocking ACE but can also cause side effects such as itching, taste alterations, coughing, and hyperkalemia. Therefore, the development of new angiotensin‐converting enzyme‐inhibitory (ACE‐I) peptides with minimal side effects has emerged as a research focus (Xiang et al. 2024). Furthermore, hypertension is frequently comorbid with Type 2 diabetes, as both conditions share overlapping metabolic pathways. Dipeptidyl peptidase IV (DPP‐IV) is a key enzyme involved in glucose metabolism, and its inhibition increases insulin secretion and improves glycemic control. Therefore, inhibitors of DPP‐IV have shown therapeutic potential in the treatment of type 2 diabetes (Chourasia 2023). As a result, dietary bioactive peptides may contribute to preventive nutritional strategies through their potential ACE‐I activity and predicted DPP‐IV‐related interactions.

Dairy products, particularly cheeses, are recognized as rich sources of bioactive peptides released during proteolytic processes driven by enzymatic hydrolysis or microbial fermentation. However, the innovation of hybrid food matrices (combining animal and plant‐based proteins) presents a novel biotechnological approach to enhance bioactivity beyond what is possible with a single protein source (Genet et al. 2023). Cheeses are well‐recognized sources of bioactive peptides with reported ACE‐I activity and DPP‐IV‐related bioactive potential (Timón et al. 2014). Other studies have reported that bioactive peptides with ACE and DPP‐IV inhibitory properties can be found in Cheddar, Parmigiano‐Reggiano, Prato, and Kaşar cheese (Baptista et al. 2020; Cattivelli et al. 2024; Gürmeriç et al. 2024; Qiu et al. 2024). These peptides are released during proteolytic processes associated with enzymatic hydrolysis and microbial fermentation. Cheeses produced with cow's milk and soybean beverage mixtures have been reported to show ACE‐I activity. The addition of soybean beverage as a plant‐based protein source provides a synergistic effect (Ali et al. 2017; Wang et al. 2015). As a result, cheeses with various peptide profiles can have potential health‐promoting effects (Undhad Trupti et al. 2021). Furthermore, cheese production can use starter cultures containing various bacterial strains, individually or in mixtures (Blaya et al. 2018). Strains such as *Lactobacillus helveticus* and *Lactobacillus delbrueckii* are preferred due to their strong and specific proteolytic activity. Moreover, studies have shown that these two cultures can produce ACE‐I peptides (Wu et al. 2022, 2023, 2024). While these strains have been studied in pure dairy matrices, their proteolytic efficiency and specific cleavage patterns in mixed dairy‐soy matrices remain insufficiently explored. Specifically, it remains unclear whether animal and plant proteins can generate a synergistic peptidomic profile during co‐fermentation. Such a profile may enhance specific bioactivities beyond those achievable with a single protein source. Moreover, limited information is available on the identity and predicted enzyme‐binding behavior of soybean‐derived peptides generated within cheese matrices.

To address this gap, we hypothesized that co‐fermentation of cow's milk and soybean beverage with proteolytic starter cultures, particularly *Lactobacillus helveticus* , would promote the release of a more diverse peptide profile from the hybrid dairy–soy matrix. We further postulated that soybean fortification could contribute additional cryptic peptide sequences with ACE‐I activity and predicted DPP‐IV‐related bioactivity. Therefore, this study aimed to investigate the effects of four soybean beverage concentrations and two starter culture combinations on the peptidomic profiles and bioactive potential of hybrid cheeses during ripening. By integrating in vitro ACE‐I assays, peptidomic profiling based on liquid chromatography–tandem mass spectrometry (LC–MS/MS), bioinformatics screening, and molecular docking, this research provides insight into the peptide‐level mechanisms underlying the functional potential of dairy‐soy hybrid cheeses.

## 2 Materials and Methods

### 2.1 Soybean, Soybean Beverage and Cow Milk

Raw cow's milk (13.00%–13.70% total solids and pH 6.58–6.85) was supplied by a dairy plant in Trabzon, Türkiye. A local soybean variety, Samsoy ( *Glycine max* (L.) Merr.), was obtained from the Black Sea Agricultural Research Institute in Samsun, Türkiye.

The soybean beverage was produced following the method described by Li et al. (2013) with modifications to standardize its total solid content equivalent to that of the cow's milk. To prepare the cheese milk formulations, the standardized soybean beverage was mixed with cow's milk at four different mass ratios (w/w): 0% (control), 13%, 26%, and 39%. The cheese manufacturing process was carried out in two independent trials.

The study employed a factorial design involving four soybean concentrations and two different starter culture combinations. All subsequent analytical measurements were performed in triplicate. The resulting cheese samples were coded as follows:

- Soybean ratios: The prefixes 0, 13, 26, and 39 indicate the percentage of soybean beverage in the mixture.
- Starter cultures: The suffixes H and A denote cheeses produced with *Lactobacillus helveticus* and *Lactobacillus acidophilus* based culture combinations, respectively. For example, 39H represents cheese made with 39% soybean beverage and *L. helveticus* culture.

### 2.2 Bacterial Cultures and Growth Conditions

The study utilized three *Lactobacillus* strains ( *Lactobacillus casei* ATCC 393, *Lactobacillus acidophilus* ATCC 4356, and *Lactobacillus helveticus* ATCC 15009) and two *Lactococcus* strains ( *Lactococcus lactis* ATCC 19435 and *Lactococcus cremoris* ATCC 19257) (Microbiologics, Kwikstik). The specific starter culture combinations (codified as H and A) used for cheese production are detailed in Table S1. Stock cultures were activated by inoculating them into MRS broth (for *Lactobacillus* strains) and M17 broth (for *Lactococcus* strains), followed by overnight incubation at 37°C and 32°C–33°C, respectively. To prepare the bulk starter for cheesemaking, the activated cultures were sub‐cultured twice in sterile reconstituted skim milk (10% solids, w/v). The final bulk starter was prepared by inoculating sterile skim milk with the active cultures and incubating until the desired cell density and acidity were achieved prior to cheese production.

### 2.3 Cheesemaking Procedure

Cheese production was carried out using a modified version of the method described by Erkaya and Şengul (2015). First, the raw cow's milk was pasteurized at 68°C for 15 min and cooled to 33°C while the soybean beverage was pasteurized at 85°C–90°C for 10 min and cooled to the same temperature. The pasteurized cow's milk and soybean beverage were mixed at varying ratios to obtain cheese milk formulations containing 0%, 13%, 26%, and 39% soybean beverage (w/w). Each mixture was then inoculated with the specific starter culture combination (H or A) at a rate of 3% (v/v). The inoculated mixtures were allowed to ripen at 33°C until the pH dropped to approximately 6.0. Following acidification, coagulation was induced by the addition of CaCl_2_‐enriched microbial rennet (Mucor miehei, 1:16,000 strength, Mayasan Food Co., Istanbul, Türkiye) for the casein fraction, and CaSO_4_·2H_2_O (Merck, 0.7%, w/v) to facilitate the coagulation of soybean proteins. The mixtures were incubated at 33°C for 45 min to form a firm curd. The resulting curds were cut, drained, and pressed into molds. The cheese blocks were vacuum‐packaged and ripened at 4°C for 45 days.

### 2.4 Proteolytic Hydrolysis

Cheese samples were subjected to proteolytic hydrolysis using a modified simulated gastrointestinal digestion model (Martini et al. 2020). Briefly, 10 g of the cheese sample was homogenized with 10 mL of distilled water and pre‐incubated at 37°C for 5 min (100 rpm). The two‐stage digestion commenced with a simulated gastric phase, where the homogenate's pH was adjusted to 2.0 with 6 M HCl, followed by the addition of 20 mL of pepsin solution (Sigma‐Aldrich, P7000 and containing 4000 U mL^−1^ of porcine pepsin) and incubation at 37°C for 2 h. For the subsequent intestinal phase, the pH was neutralized to 7.0 using 6 M NaOH, and 40 mL of pancreatin solution (Sigma‐Aldrich, P1750 and containing pancreatin 200 U mL^−1^ based on trypsin activity) was added for an additional 2 h of incubation at 37°C. Finally, the intestinal digestion was terminated by heating the samples at 95°C for 10 min, followed by rapid cooling in an ice bath. The clear hydrolysates were collected via centrifugation at 10,000 × *g* for 20 min at 4°C.

### 2.5 Protein Assay

The concentration of water‐soluble proteins (WSP) in the clear supernatants obtained from the simulated gastrointestinal digestion was determined using the Lowry method (Lowry et al. 1951). Bovine serum albumin (BSA) was utilized as the standard protein to construct the calibration curve. The absorbance values were measured at 660 nm using a UV–Vis spectrophotometer, and the results were expressed as mg BSA equivalent/mL of hydrolysate.

### 2.6 Assessment of Proteolytic Activity

The degree of proteolysis in the cheese hydrolysates was determined using the *o*‐phthalaldehyde (OPA) spectrophotometric assay as described by Ahtesh et al. (2018), with modifications. The OPA reagent was prepared fresh daily. Briefly, 40 mg of OPA (Sigma‐Aldrich) was dissolved in 1 mL of methanol and added to 25 mL of 100 mM disodium tetraborate buffer. Subsequently, 2.5 mL of 20% (w/w) sodium dodecyl sulfate (SDS) and 100 μL of β‐mercaptoethanol were incorporated. The final volume was adjusted to 50 mL with distilled water. For the analysis, 30 μL of the cheese hydrolysate (or standard) was mixed with 1 mL of the OPA reagent. The mixture was incubated at room temperature for 2 min. The absorbance was measured at 340 nm using a UV–Vis spectrophotometer. A standard calibration curve was constructed using tryptone (0.07–2.5 mg/mL), and the results were expressed as mg tryptone equivalent/mL cheese extract (*R* ^2^ > 0.9995).

### 2.7 Determination of ACE‐I Activity

The ACE‐I activity was determined according to the method developed by Cushman and Cheung (1971). The substrate solution consisted of 5 mM Hippuryl‐Histidyl‐Leucine (HHL; Sigma‐Aldrich) dissolved in 100 mM sodium borate buffer containing 300 mM NaCl (pH 8.3). For the assay, 50 μL of the peptide hydrolysate (sample) was mixed with 180 μL of the HHL substrate solution and pre‐incubated at 37°C for 5 min. The reaction was initiated by the addition of 20 μL of ACE (A‐6778, Sigma‐Aldrich). The mixture was incubated at 37°C for 15 min. The enzymatic reaction was terminated by adding 250 μL of 1 M HCl. The released hippuric acid was extracted by adding 1.7 mL of ethyl acetate. The phases were separated by centrifugation, and 1.0 mL of the upper organic layer was transferred to a clean tube and evaporated to dryness in a boiling water bath (100°C). The residue was dissolved in 2 mL of distilled water, and the absorbance was measured at 228 nm using a UV–Vis spectrophotometer. Captopril was used as a positive control, while distilled water served as the negative control. The percentage of ACE‐I was calculated using Equation (1):

(1) $$\mathrm{ACE}-I\left(\%\right)=\left[\left(A-B\right)/A\right]\times 100$$

Here, *A*: absorbance in the presence of ACE and HHL but without ACE inhibitor (control) and *B*: absorbance in the presence of ACE, HHL, ACE inhibitor (test sample).

### 2.8 Peptidomic Profiling by LC–MS/MS and In Silico Analysis

The peptidomic analysis was performed using a modified version of the method described by Barbu et al. (2021). Peptide separation and identification were carried out using a Waters ACQUITY UPLC I‐CLASS PLUS LC System coupled with a Xevo TQ‐XS Mass Spectrometer (Waters Corp., Milford, MA, USA). Chromatographic separation was achieved on a BEH C18 column (2.1 mm × 50 mm, 1.7 μm) maintained at 40°C, with UV detection monitored at 214 nm. The mobile phases consisted of water containing 1% (v/v) formic acid (Eluent A) and acetonitrile containing 1% (v/v) formic acid (Eluent B). The flow rate was set to 0.5 mL/min. The gradient elution program was applied as follows: initial hold at 15% B for 5 min; linear increase to 25% B from 5 to 8 min; linear increase to 50% B from 8 to 10 min; increase to 85% B from 10 to 12 min; followed by reequilibration at 15% B from 12 to 25 min.

Mass spectrometry (MS) analysis was performed using an electrospray ionization (ESI) source in positive mode. The instrument operated in multiple reaction monitoring (MRM) and mass scan modes. The optimized conditions were as follows: capillary voltage 0.5 kV, cone voltage 60 V, collision energy 20–40 eV, source temperature 120°C, desolvation temperature 300°C, and desolvation gas flow rate 800 L/h.

Peptide sequences were matched against protein entries from the UniProt and Protein Data Bank databases using Skyline and PEAKS Studio X+ software (www.bioinfor.com/peaks‐studio and https://skyline.ms/project/home/software/Skyline/begin.view). To ensure the accuracy of peptide identification, a false discovery rate threshold of ≤ 1% was applied alongside appropriate mass error tolerances for precursor and fragment ions. Additionally, the mass error tolerance was set to < 10 ppm for precursor ions and < 0.05 Da for fragment ions. Peptide sequences were validated based on a high Average Local Confidence (ALC) score (≥ 80%) derived from the PEAKS Studio software. In silico validation of peptide bioactivity was performed based on the PeptideRanker score (Minkiewicz et al. 2008). To manage the large dataset generated by LC–MS/MS (102 identified sequences) and to ensure that the subsequent in silico docking simulations were biologically relevant, a systematic screening strategy was implemented using the PeptideRanker server. The rationale behind using this tool was to filter out nonfunctional fragments and isolate peptides with the highest mathematical probability of exhibiting physiological bioactivity. PeptideRanker scores were used to prioritize peptides with high predicted general bioactivity. A threshold score of > 0.50 was primarily considered to identify peptides with relatively high predicted bioactive potential. In addition, peptides annotated in BIOPEP‐UWM or identified as ACE‐ or DPP‐IV‐related sequences were also considered for docking when relevant to the study hypothesis. This combined screening strategy allowed the selection of both high‐scoring peptides and database‐supported enzyme‐related sequences for subsequent ACE and DPP‐IV molecular docking analysis.

### 2.9 Molecular Docking Studies

Molecular docking studies were performed to obtain predictive structural insight into the potential interactions between the selected peptides and the target enzymes. The crystal structures of ACE (PDB ID: 1O8A) and DPP‐IV (PDB ID: 5Y7K) were retrieved from the RCSB Protein Data Bank. Prior to docking, water molecules and co‐crystallized ligands were removed, and the receptor structures were prepared for docking analysis. Protein–peptide docking simulations were carried out using the HPEPDOCK server (http://huanglab.phys.hust.edu.cn/hpepdock/) (Zhou et al. 2018). HPEPDOCK is a web‐based tool designed to predict peptide binding modes to target proteins through a hierarchical docking algorithm and accounts for peptide flexibility during the docking process. The docking outputs were evaluated based on predicted docking scores and the visual inspection of peptide–enzyme complexes. More negative docking scores were interpreted as indicating more favorable predicted binding poses. In addition, noncovalent interactions, including hydrogen bonds and hydrophobic contacts between peptide ligands and amino acid residues of the target enzymes, were analyzed to support the interpretation of potential binding mechanisms. These docking results were used as comparative and predictive indicators rather than direct confirmation of biological activity.

### 2.10 Statistical Analysis

The statistical analysis of the in vitro results was performed using the SPSS 20.0 software package (SPSS Inc., Chicago, IL). All data were expressed as the mean ± standard deviation (SD), calculated from the two independent cheese manufacturing trials and their respective triplicate analytical measurements. A general linear model and ANOVA were utilized to evaluate the main effects of the independent variables (soybean beverage concentration and starter culture type) on the dependent variables, which included WSP content, degree of proteolysis (OPA), and ACE‐I activity. The statistical differences among the eight distinct experimental cheese formulations were determined using Duncan's multiple range test, with the significance level set at *p* < 0.05.

## 3 Results and Discussion

### 3.1 In Vitro Assays of Cheese Proteolytic Hydrolysates

#### 3.1.1 WSP Content

The concentration of WSP in the gastrointestinal hydrolysates varied depending on the cheese formulation, ranging from 4.92 to 16.81 mg BSA/mL (Table 1). The control samples produced from cow's milk (0H and 0A) exhibited the highest WSP levels (14.69 and 16.81 mg BSA/mL, respectively). This result indicates that cow's milk proteins were more readily solubilized during simulated gastrointestinal digestion. In contrast, a progressive decline in WSP concentration was observed as the proportion of soybean beverage increased. The samples formulated with the highest soybean concentration (39H and 39A) displayed the lowest WSP values (4.92 and 6.79 mg BSA/mL, respectively) (*p* < 0.05) (Table 1). This inverse relationship suggests that bovine milk proteins, particularly caseins, were more susceptible to solubilization during simulated digestion. Conversely, soybean globular proteins, including glycinin and β‐conglycinin, may have been less accessible due to the formation of insoluble aggregates or different hydrolysis kinetics. Despite these variations, the WSP values obtained in this study are consistent with those reported in the literature. For instance, Banihashemi et al. (2020) reported WSP levels ranging from 6.2 to 16.0 mg/mL in traditional Iranian Koopeh cheese, while (Meira et al. 2012) found values between 6.5 and 15.0 mg/mL in sheep cheeses ripened for 60 days.

**TABLE 1:** WSP content, proteolysis level, and ACE‐I activity of proteolytically hydrolyzed cheese samples.

Sample codes	mg BSA/mL	mg tryptone/mL	ACE‐I (%)
0A	16.81 ± 0.41^a^	7.72 ± 0.56^f^	71.22 ± 0.63^b^
0H	14.69 ± 0.41^b^	10.29 ± 0.71^e^	72.91 ± 1.02^ab^
13A	10.98 ± 0.83^d^	8.17 ± 0.42^f^	45.14 ± 0.32^e^
13H	10.34 ± 0.91^d^	11.22 ± 0.25^d^	52.23 ± 2.81^d^
26A	13.92 ± 1.33^bc^	11.56 ± 0.34^d^	45.56 ± 0.62^e^
26H	13.02 ± 1.56^c^	15.50 ± 0.14^b^	59.48 ± 0.53^c^
39A	6.79 ± 0.64^e^	12.82 ± 0.21^c^	53.47 ± 0.64^d^
39H	4.92 ± 0.08^f^	17.72 ± 0.28^a^	74.35 ± 2.19^a^

*Note:* H: *Lactobacillus helveticus* combination culture; A: *Lactobacillus acidophilus* combination culture; 0: 100% cow's milk cheese; 13: 13% soybean beverage + 87% cow's milk cheese; 26: 26% soybean beverage + 74% cow's milk cheese; 39: 39% soybean beverage + 61% cow's milk cheese. Different superscript letters within the same column indicate significant differences at *p* < 0.05.

Abbreviations: ACE‐I, angiotensin‐converting enzyme inhibition; BSA, bovine serum albumin; OPA, *o*‐phthalaldehyde assay; WSP, water‐soluble protein.

#### 3.1.2 Proteolytic Activity

The degree of proteolysis, measured by the OPA assay, revealed a contrasting trend compared with WSP content. While WSP mass decreased with soybean addition, the proteolytic activity significantly increased (Table 1). The peptide content varied between 7.72 and 17.72 mg tryptone/mL after 45 days of ripening. Notably, cheeses fermented with the *L. helveticus* combination (H code) exhibited higher proteolysis values compared to those with *L. acidophilus* (A code). The highest proteolytic activity was recorded in the 39H sample (17.72 ± 0.28 mg/mL), while the lowest was in the 0A sample (7.72 ± 0.56 mg/mL). This trend suggests that the proteolytic system of *L. helveticus* was particularly effective in hydrolyzing the complex protein matrix of the hybrid soybean‐dairy cheeses. These findings align with Yousefi et al. (2021) and support the view that *L. helveticus* possesses a stronger proteolytic capacity than other lactic acid bacteria used in cheese ripening.

#### 3.1.3 ACE‐I Activity and Structure–Function Relationship

The ACE‐I activity of the hydrolysates showed a clear dependency on both the soybean concentration and the starter culture. As detailed in Table 1, the inhibition percentage ranged widely from 45.14% to 74.35%. The control samples (0H and 0A) showed high activities of 72.91% and 71.22%, respectively. However, the incorporation of soybean beverage caused a fluctuation; samples 13A (45.14%) and 13H (52.23%) exhibited lower inhibition rates. Furthermore, the 39H sample achieved the highest ACE‐I activity of 74.35% (*p* < 0.05). In contrast, the 39A sample showed a moderate activity of 53.47%.

A critical comparison reveals an interesting phenomenon: while the 39H sample had the lowest total WSP content (4.92 mg/mL), it possessed the highest ACE‐I activity. This inverse relationship indicates that total soluble protein mass is not a reliable predictor of bioactivity in these hybrid matrices. Instead, ACE‐I activity appears to be primarily driven by the qualitative peptide profile, including specific amino acid sequences, molecular size, and the presence of hydrophobic or aromatic residues. Large, partially hydrolyzed protein fragments may contribute heavily to WSP mass but may not necessarily possess structural features required for ACE inhibition. Conversely, co‐fermentation by *L. helveticus* in the 39H matrix likely accelerated the breakdown of larger proteins into smaller peptide fragments with higher predicted bioactive potential. Consequently, although the total measurable protein mass decreased, the accumulation of specific short‐chain peptides, including soybean‐derived sequences such as DQMPRRF and SGRAIL identified in the peptidomic analysis, may have contributed to the enhanced ACE‐I activity.

This result is significant when compared to previous studies. For instance, Erkaya and Şengul (2015) reported that the ACE‐I activities of water‐soluble extracts of feta cheese ripened for 120 days ranged between 15.62 and 65.06. They also found that the presence of probiotic *L. acidophilus* in the starter culture increased ACE‐I activity. The highest activity observed in our study is three times higher than the lowest activity reported by them. In addition, Hao et al. (2023) emphasized that *L. helveticus* has a stronger capacity than other lactic acid bacteria to promote the release of antihypertensive peptides in cheese. Our results align with recent investigations on Turkish cheeses and kefir, which suggest that the strategic use of proteolytic *Lactobacillus* species can influence the accumulation of low‐molecular‐weight fractions and affect the final functional efficacy (Altuntas and Altuntas 2025; Atalay and Şahingil 2022). Ultimately, the advantage of the present model lies in complementing dairy hydrolysis with plant‐derived protein degradation. As reported by Xu et al. (2021), the gastrointestinal digestion of soybean proteins can release peptide fragments with ACE‐I potential. The introduction of 39% soybean beverage expanded the structural spectrum of hydrolyzable precursor sequences. This expansion allowed the proteolytic system of *L. helveticus* to generate a diverse peptide pool with enhanced ACE‐I potential.

### 3.2 Peptidomic Profiling by LC–MS/MS

The LC–MS/MS analysis provided a comprehensive insight into the peptide composition of the cheese samples and revealed how soybean fortification and starter culture type influenced proteolysis. A total of 102 peptide sequences were identified across the eight cheese formulations. Although the pure dairy control fermented with *L. helveticus* (0H) showed a higher soluble protein content than the 39H sample (Table 1), the chromatographic profile of 39H contained distinct signals corresponding to both dairy‐ and soybean‐derived peptides. This indicates that soybean incorporation did not simply increase the total soluble protein fraction, but rather altered the composition of the peptide pool generated during digestion and ripening. This is consistent with previous findings that soybean proteins can serve as versatile precursors for ACE‐I fragments during enzymatic hydrolysis (Xu et al. 2021).

The functional relevance of this altered peptide profile is supported by the high ACE‐I activity observed in the 39H sample. This sample also showed the highest proteolysis value, measured as tryptone equivalents (17.72 mg/mL), suggesting that the proteolytic system of *L. helveticus* promoted the conversion of larger protein fractions into smaller peptide fragments and free amino acids (Altuntas and Altuntas 2025; Atalay and Şahingil 2022). Such fragments may possess different sequence compositions and predicted interaction patterns, allowing them to interact with different regions of ACE and DPP‐IV binding pockets, as suggested by docking‐based studies (Gu et al. 2023). Therefore, the enhanced ACE‐I activity of 39H appears to be more closely related to peptide composition and proteolytic specificity than to total soluble protein content alone.

The total ion chromatograms further showed differences between the starter culture combinations (Figures S1–S8). The ion spectra of 0H cheese displayed higher relative peak intensities compared to 0A. This finding suggests the enhanced proteolytic efficiency of *L. helveticus* compared to *L. acidophilus* (Hao et al. 2023). Furthermore, the soybean‐fortified samples, particularly 39H, showed a distinctive profile with dominant ion signals in the low molecular weight region, such as those around 354 *m*/*z*, and specific high‐mass peaks in the 1600–1950 *m*/*z* range. These spectral patterns may reflect the accumulation of peptide fragments with diverse molecular sizes. While Meira et al. (2012) demonstrated that specific hydrophobic residues contribute to ACE‐I potency in traditional dairy matrices, Tang et al. (2025) reported that soybean‐derived peptides with terminal hydrophobic domains may interact favorably with ACE catalytic sub‐pockets. Therefore, the accumulation of hydrophobic peptide fragments in the 39H sample may provide a plausible molecular explanation for its high ACE‐I activity. This suggests that the enhanced bioactivity of the 39H matrix may be associated with dual‐source peptide diversity generated from both dairy and soybean proteins.

### 3.3 Identification and Functional Characterization of Peptides

LC–MS/MS analysis coupled with bioinformatics processing led to the identification of 102 unique peptide sequences across the eight cheese formulations. These peptides were categorized based on their precursor proteins: 74 peptides originated from bovine milk proteins, while 28 peptides were derived exclusively from soybean proteins in the soybean‐fortified samples. The identified sequences were cross‐referenced with the BIOPEP‐UWM database to determine their potential biological activities. The analysis demonstrated a wide spectrum of peptides characterized by known ACE‐I fragments and predicted or database‐annotated DPP‐IV‐related bioactive fragments. The distribution of these peptides varied depending on the soybean concentration and the starter culture.

#### 3.3.1 Peptides Derived From Bovine Milk Proteins

The hydrolysis of bovine milk proteins resulted in a wide range of bioactive peptides, predominantly derived from casein fractions (α_s1_‐, α_s2_‐, β‐, and κ‐casein) and whey proteins (α‐lactalbumin, β‐lactoglobulin, and lactoferrin). The complete inventory of these peptides, along with their specific distribution across the eight cheese formulations is provided in Table S2.

##### 3.3.1.1 Casein‐Derived Peptides

Peptides originating from α_s1_‐casein ranged from 3 to 14 amino acids in length (Table S2). A recurring core sequence, FFVAPFPEVFGK, was detected in all cheese varieties. This sequence contains the potent ACE‐I fragment FFVAP, which has been previously reported in Prato cheese (Baptista et al. 2020). Furthermore, the ENLLRF sequence and its extended forms, including LNENLLLRFFVAPFPEVFG, were identified. These findings suggest a conserved proteolytic cleavage pattern catalyzed by *L. helveticus* and *L. acidophilus* proteases.

Regarding α_s2_‐casein, the peptides exhibited a diverse structural profile with molecular weights ranging from 375.51 Da (MKP) to 1368.53 Da (ALNEINQFYQK), as detailed in Table S2. The VPITPTL peptide, known for its DPP‐IV inhibitory activity (Helal and Tagliazucchi 2023), was identified in the samples. Furthermore, peptides such as AMKPWIQPK and FALPQYLK, which Martini et al. (2020) detected in Parmigiano Reggiano only after 24 months of ripening, were released in the present cheese samples within 45 days.

The β‐casein fraction also proved to be a rich source of bioactive peptide sequences (Table S2). The opioid and ACE‐I peptide DKIHPF previously found in Spanish blue cheese (Sánchez‐Rivera et al. 2014) was present in all samples (Table S2). Moreover, the multifunctional peptide AVPYPQR, known for its antioxidant and antimicrobial properties (Yang et al. 2021), was released in both ‘H’ and ‘A’ series cheeses.

Finally, peptides derived from κ‐casein (3–8 amino acids) were associated with antioxidant, ACE‐I, and DPP‐IV‐related bioactive annotations (Table S2). A notable finding is the detection of the ACE‐I peptide VLSRYP, which was previously isolated from kefir fermented with *Kluyveromyces marxianus* (Li et al. 2015). Despite the differences in the fermentation matrix (kefir vs. cheese), the release of the same bioactive sequence highlights the importance of the common protein substrate (cow's milk).

##### 3.3.1.2 Whey‐Derived Peptides

The release of whey‐derived peptides appeared to be modulated by the soybean concentration. Peptides derived from α‐lactalbumin, such as LAHKALCSEKL, were abundant in samples with low soybean content (0% and 13%) but were absent or reduced in samples containing > 26% soybean beverage (Table S2). A similar trend was observed for β‐lactoglobulin‐derived peptides, which were restricted to the low‐soybean formulations (Table S2). This suggests that high soybean concentrations might protect whey proteins from enzymatic attack or alter the enzyme‐substrate ratio. Conversely, lactoferrin‐derived peptides, such as GILRP and PYKLRP, were detected in the high‐soybean (39%) samples (Table S2).

#### 3.3.2 Peptides Derived From Soybean Proteins

A novel aspect of this study is the identification of 28 soybean‐derived peptides in the fortified cheese samples (26H, 39H, 26A, 39A) (Table S2). These peptides exhibited ACE‐I activity‐related annotations and predicted DPP‐IV‐related bioactive potential. Several sequences identified in this study represent novel peptides that have not been previously documented in fermented cheese matrices. For instance, the peptides NANSIIYAL, AGNQEQEF, RKNAMF, and EYVSF, derived from glycinin G2, were detected in the soybean‐fortified samples (Table S2). The absence of these sequences in existing literature highlights the unique proteolytic environment created by the co‐fermentation of soybean and dairy proteins with *L. helveticus* . Furthermore, Voss et al. (2019) reported the peptide DQMPRRF while investigating the effects of enzymatic hydrolysis and heat treatment on the functional properties of soybean meal. To our knowledge, the present study is the first to report the DQMPRRF sequence in a fermented cheese matrix. Additionally, the QSKPNTIL peptide, previously associated with heat‐treated soybeans (Zhang et al. 2023), was released from β‐conglycinin. The detection of these soybean‐specific peptides is consistent with the high ACE‐I activity observed in the 39H sample. This suggests that soybean proteins may serve as important precursors of functional peptides in hybrid cheeses.

#### 3.3.3 Structure–Activity Relationships and Functional Relevance of Identified Peptides

The notable differences in ACE‐I activity and predicted DPP‐IV‐related bioactivity across the formulations were not likely attributable solely to peptide quantity. Instead, the superior bioactivity of the 39H sample appears to be associated with the specific amino acid architecture of the sequences released during co‐fermentation. It is widely acknowledged that ACE is a zinc‐metalloproteinase that exhibits a strong preference for competitive inhibitors possessing specific hydrophobic or bulky aromatic amino acid residues at their C‐terminal positions, such as proline (P), phenylalanine (F), tryptophan (W), or tyrosine (Y) (Tang et al. 2025; Xu et al. 2021).

In the hybrid matrix, the identification of the casein‐derived sequence FFVAPFPEVFGK and the novel soybean‐derived sequence DQMPRRF appears to confer a structural advantage. The FFVAPFPEVFGK sequence harbors the active encrypted fragment FFVAP. The accumulation of these specific residues may provide favorable spatial compatibility with ACE binding sub‐pockets, specifically the S1, S1′, and S2′ sites. Similarly, the soybean peptide DQMPRRF contains C‐terminal residues that may support hydrophobic interactions within the ACE binding region (Xu et al. 2021). For DPP‐IV, the functional relevance of the identified soybean peptides such as QRQF and MQVPVL is related to their predicted structural features. DPP‐IV specifically cleaves N‐terminal dipeptides with proline or alanine, and sequences rich in branched‐chain or hydrophobic residues may act as alternative substrates or competitive inhibitors (Gu et al. 2023). The structural diversity achieved by combining dairy and plant substrates expands the spectrum of hydrolyzable precursor proteins, enabling *L. helveticus* proteolytic systems to generate diverse peptide sequences. Rather than relying on a single mechanism, the co‐fermented hybrid matrix may provide diverse dairy‐ and soybean‐derived peptide sequences. These peptides may facilitate complementary enzyme interactions by interacting with distinct functional regions of metabolic enzymes (Gu et al. 2023; Rosa et al. 2026).

### 3.4 Molecular Docking and Interaction Analysis

Prior to molecular docking simulations, a systematic screening strategy was applied to select candidate peptides from the identified peptide pool. Rather than relying on random selection, the predicted bioactivity potential of the peptide sequences was evaluated using the PeptideRanker server and BIOPEP‐UWM annotations. A PeptideRanker threshold of > 0.50 was used to identify peptides with high predicted general bioactivity (Table S3). In addition, selected ACE‐ or DPP‐IV‐related peptides with database‐supported functional relevance were also included in the docking analysis, even when their PeptideRanker scores were below this threshold. This combined screening strategy enabled the docking study to focus on peptide sequences with either high predicted general bioactivity or specific enzyme‐related relevance toward ACE and DPP‐IV.

Molecular docking simulations were performed against ACE (PDB: 1O8A) and DPP‐IV (PDB: 5Y7K) using the HPEPDOCK server. The docking scores of the selected peptides were comparatively evaluated based on their HPEPDOCK ranking values. Since molecular docking provides predictive structural information rather than direct confirmation of biological activity, the docking results were interpreted as supportive indicators complementing the in vitro findings.

For ACE, the docking analysis suggested that selected milk‐ and soybean‐derived peptides exhibited favorable predicted binding conformations, as indicated by their comparatively negative HPEPDOCK scores (Tables 2 and 3). Among the milk‐derived peptides, FFVAPFPEVFGK showed the most favorable first‐ranked docking score, followed by LAYFYP and AYFYPEL (Table 2). Among the soybean‐derived peptides, DQMPRRF showed the most favorable predicted docking score, followed by HWANL and RKNAMF (Table 3). These results suggest potentially favorable peptide‐enzyme interactions and are consistent with docking patterns previously reported for bioactive peptides derived from dairy and plant protein hydrolysates (Rosa et al. 2026). Therefore, the docking findings provide structural support for the ACE‐I potential observed in the biochemical assays, while not independently confirming biological activity.

**TABLE 2:** Molecular docking scores of selected milk‐derived peptides with predicted ACE‐I potential against ACE.

PDB code	Peptides hydrolyzed from cow milk				
1O8A	ALPM	AMKPW	AYFYPEL	FFVAPFPEVFGK	LAYFYP
Rank	Docking scores				
1	−135.913	−199.721	−239.807	−268.170	−244.282
2	−133.121	−192.780	−239.061	−257.049	−241.808
3	−129.017	−192.224	−238.160	−246.048	−240.705
4	−128.355	−192.029	−235.719	−226.647	−240.136
5	−128.330	−190.812	−230.183	−219.942	−237.577
6	−125.518	−190.196	−229.093	−217.589	−237.303
7	−125.431	−189.584	−226.285	−216.213	−237.131
8	−124.533	−188.800	−225.869	−212.331	−236.315
9	−123.722	−187.488	−224.523	−210.832	−234.909
10	−123.647	−186.189	−224.188	−210.469	−232.343

*Note:* Docking scores were obtained using the HPEPDOCK server. More negative scores indicate more favorable predicted binding poses. These values should be interpreted as comparative and predictive docking indicators rather than direct experimental binding free energies or confirmation of biological activity.

Abbreviations: ACE, angiotensin‐converting enzyme; HPEPDOCK, hierarchical flexible peptide docking; PDB, Protein Data Bank.

**TABLE 3:** Molecular docking scores of selected soybean‐derived peptides with predicted ACE‐I potential against ACE.

PDB code	Peptides hydrolyzed from soybean beverage				
1O8A	DQMPRRF	RKNAMF	SGRAIL	HWANL	QRQF
Rank	Docking scores				
1	−230.682	−214.065	−182.486	−227.966	−197.554
2	−216.817	−212.739	−176.930	−223.274	−191.886
3	−214.400	−208.013	−174.427	−220.650	−181.563
4	−212.314	−207.391	−174.006	−216.515	−180.537
5	−211.924	−207.141	−170.848	−215.866	−178.256
6	−210.443	−206.133	−169.728	−215.180	−178.226
7	−208.857	−204.259	−168.874	−215.073	−177.912
8	−207.856	−204.188	−168.230	−214.892	−177.178
9	−206.291	−202.594	−162.628	−212.842	−176.463
10	−205.518	−202.558	−162.510	−209.731	−176.302

*Note:* Docking scores were obtained using the HPEPDOCK server. More negative scores indicate more favorable predicted binding poses. These values should be interpreted as comparative and predictive docking indicators rather than direct experimental binding free energies or confirmation of biological activity.

Abbreviations: ACE, angiotensin‐converting enzyme; HPEPDOCK, hierarchical flexible peptide docking; PDB, Protein Data Bank.

**FIGURE 1:**
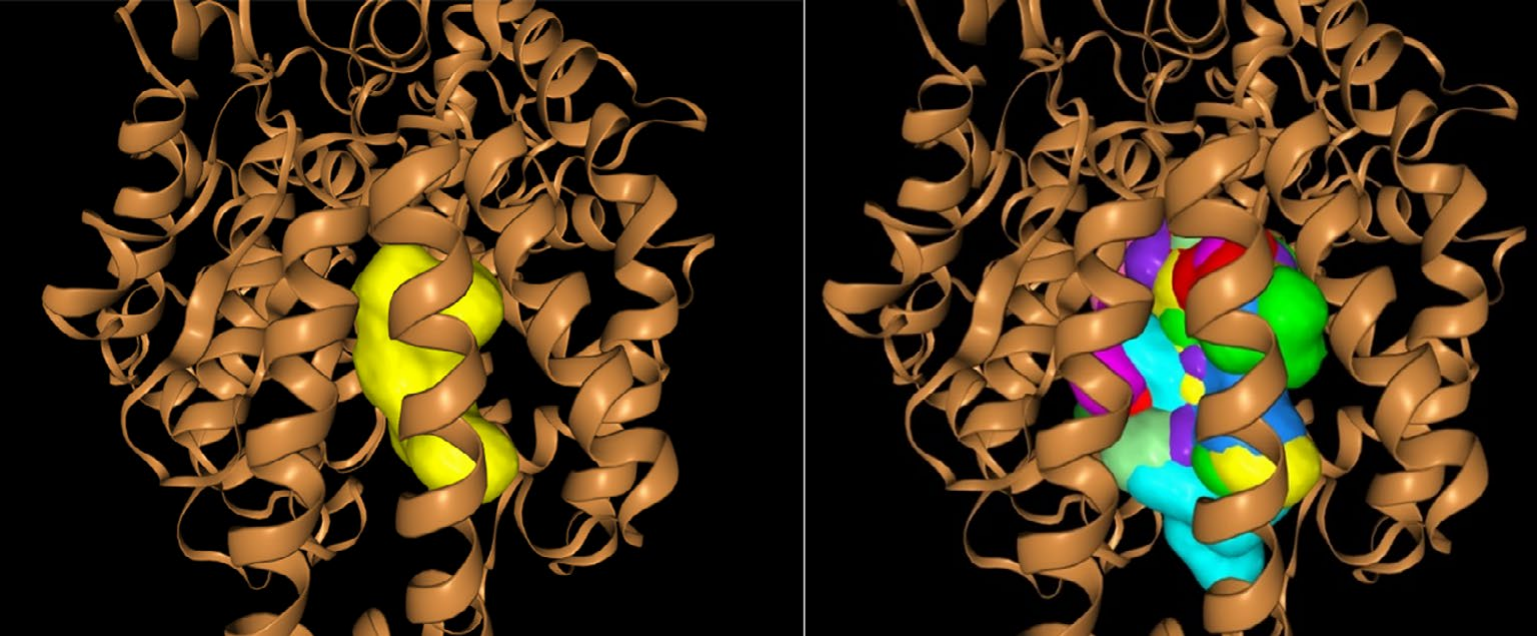
Predicted molecular docking poses of selected milk‐ and soybean‐derived peptides within the ACE structure (PDB ID: 1O8A). The enzyme is shown in ribbon representation, while docked peptides are displayed as colored molecular surfaces. These visualizations represent predicted binding conformations obtained using HPEPDOCK and should be interpreted together with the docking scores in Tables 2 and 3.

At the structural level, visual analysis of the docking poses (Figure 1) suggested that the soybean‐derived peptide DQMPRRF may be positioned within the ACE binding cavity rather than interacting only with the external enzyme surface. This predicted orientation may contribute to steric interference with substrate access and is consistent with mechanisms previously proposed for soybean‐derived ACE‐I peptides (Tang et al. 2025). Previous molecular dynamics studies have emphasized that interactions near the ACE catalytic region, including the zinc‐coordinating environment involving His383, His387, and Glu411, can be relevant for ACE inhibition by food‐derived peptides (De Oliveira et al. 2021). However, in the present study, the docking results should be interpreted as predicted binding poses only. Further molecular dynamics simulations and experimental validation would be required to confirm whether these peptides directly interact with zinc‐coordinating residues or impair catalytic activity.

**FIGURE 2:**
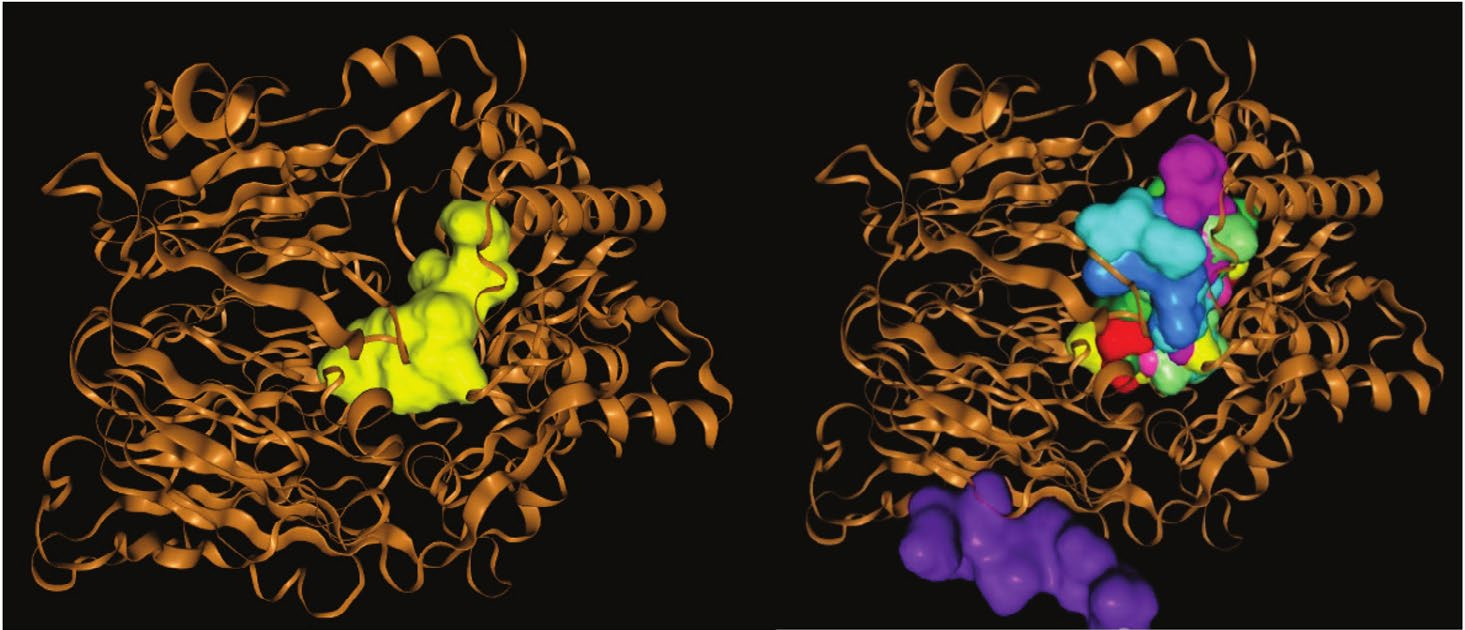
Predicted molecular docking poses of selected soybean‐derived peptides within the DPP‐IV structure (PDB ID: 5Y7K). The enzyme is shown in ribbon representation, while docked peptides are displayed as colored molecular surfaces. These visualizations illustrate predicted peptide positioning within the enzyme binding region and should be interpreted as supportive structural indicators of potential DPP‐IV‐related bioactivity.

For DPP‐IV, the soybean‐derived peptides QRQF and MQVPVL showed favorable docking scores and predicted positioning within the enzyme binding region (Table 4). QRQF exhibited the most favorable first‐ranked docking score among the selected soybean‐derived peptides, followed by MQVPVL and QSKPNTIL. Visual inspection of the docking complexes (Figure 2) suggested that these peptides may be accommodated within or near the DPP‐IV binding cavity. Such predicted binding behavior is consistent with docking‐based studies showing that peptide sequence composition can influence interactions within ACE and DPP‐IV binding regions (Gu et al. 2023). These findings suggest that QRQF and MQVPVL may represent promising soybean‐derived DPP‐IV‐related peptide candidates. Nevertheless, because DPP‐IV inhibitory activity was inferred from docking analysis rather than directly measured in vitro, these results should be considered predictive and hypothesis‐generating.

**TABLE 4:** Molecular docking scores of selected soybean‐derived peptides with predicted DPP‐IV‐inhibitory potential against DPP‐IV.

PDB code	Peptides hydrolyzed from soybean beverage				
5Y7K	QEGGVL	QRQF	MQVPVL	NSKAIVIL	QSKPNTIL
Rank	Docking scores				
1	−130.444	−187.151	−181.463	−165.266	−171.108
2	−129.620	−176.736	−168.826	−163.455	−170.448
3	−123.810	−176.716	−159.269	−159.417	−169.611
4	−123.761	−173.009	−158.809	−158.294	−169.185
5	−121.905	−172.755	−157.812	−155.856	−167.909
6	−121.824	−170.333	−157.736	−153.831	−167.067
7	−121.575	−168.387	−157.539	−152.759	−165.929
8	−121.284	−167.946	−156.456	−152.555	−163.358
9	−121.131	−167.315	−155.668	−151.409	−162.929
10	−121.088	−166.560	−155.578	−151.378	−162.885

*Note:* Docking scores were obtained using the HPEPDOCK server. More negative scores indicate more favorable predicted binding poses. These values should be interpreted as comparative and predictive docking indicators rather than direct experimental binding free energies or confirmation of biological activity.

Abbreviations: DPP‐IV, dipeptidyl peptidase IV; HPEPDOCK, hierarchical flexible peptide docking; PDB, Protein Data Bank.

Overall, the molecular docking results support the hypothesis that co‐fermentation of the dairy‐soy matrix, particularly in the 39H sample, promoted the release of peptides with favorable predicted interactions toward ACE and DPP‐IV. These structure‐based observations may partially explain the high ACE‐I activity observed experimentally and suggest a possible contribution of soybean‐derived peptides to the functional potential of the hybrid cheese matrix. However, additional validation, including in vitro DPP‐IV inhibition assays and molecular dynamics simulations, would be necessary to confirm the stability and biological relevance of the predicted peptide–enzyme interactions.

## 4 Conclusion

In conclusion, this study suggests that the co‐fermentation of dairy and soybean matrices with *L. helveticus* is a promising biotechnological strategy for generating functional hybrid cheeses. The combined use of in vitro ACE‐I assays, LC–MS/MS‐based peptidomic profiling, bioinformatics screening, and molecular docking provided evidence that soybean fortification altered the peptide profile and promoted the release of potentially bioactive sequences. A total of 102 peptide sequences were identified, including soybean‐derived peptides such as DQMPRRF, NANSIIYAL, and QSKPNTIL, which were detected for the first time in a cheese matrix. The 39H sample exhibited the highest ACE‐I activity, indicating that the combination of high soybean beverage concentration and *L. helveticus* fermentation may favor the generation of ACE‐I peptide profiles. Molecular docking further suggested that selected milk‐ and soybean‐derived peptides may interact favorably with ACE and DPP‐IV binding regions. However, these findings should be regarded as predictive and require further validation through molecular dynamics simulations, purified peptide assays, and in vitro DPP‐IV inhibition tests. Overall, this study highlights the potential of dairy‐soy hybrid cheeses as sustainable value‐added foods with promising bioactive peptide profiles. As a limitation and future direction, the DPP‐IV‐related findings of this study are based on in silico predictions and database annotations. Since in vitro DPP‐IV inhibition assays and in vivo validation were not performed, these results should be considered predictive. Future studies should include purified peptide validation, in vitro DPP‐IV assays, and in vivo investigations to confirm their biological relevance.

## Author Contributions

**Hasan Temiz:** writing – review and editing, supervision, funding acquisition, project administration. **Mehmet Kemal:** conceptualization, data curation, writing – original draft, visualization. **Mehtap Er Kemal:** formal analysis, investigation, methodology, project administration, validation, writing – original draft, funding acquisition.

## Funding

This work was supported by Türkiye Bilimsel ve Teknolojik Araştırma Kurumu (222O251), Ondokuz Mayis Üniversitesi (PYO.MUH.1901.18.009).

## Conflicts of Interest

The authors declare no conflicts of interest.

## Supporting information


**Figure S1:** Total ion spectrum of cheese coded 0H in LC–MS/MS.
**Figure S2:** Total ion spectrum of cheese coded 0A in LC–MS/MS.
**Figure S3:** Total ion spectrum of cheese coded 13H in LC–MS/MS.
**Figure S4:** Total ion spectrum of cheese coded 13A in LC–MS/MS.
**Figure S5:** Total ion spectrum of cheese coded 26H in LC–MS/MS.
**Figure S6:** Total ion spectrum of cheese coded 26A in LC–MS/MS.
**Figure S7:** Total ion spectrum of cheese coded 39H in LC–MS/MS.
**Figure S8:** Total ion spectrum of cheese coded 39A in LC–MS/MS.
**Table S1:** Experimental design and formulation of cheese samples enriched with soybean beverage and fermented with different starter cultures ( *L. helveticus* or *L. acidophilus* ).
**Table S2:** Comprehensive inventory and bioactivity profiles of identified peptides derived from bovine milk and soybean proteins.
**Table S3:** PeptideRanker scores of the identified peptides from the hybrid cheese matrices.

## Data Availability

The data that support the findings of this study are available in the Supporting Information of this article.
