# Supplementary material for: Development of Functional Soybean‐Enriched Cheese: Identification of Novel Angiotensin‐Converting Enzyme‐Inhibitory and Predicted Dipeptidyl Peptidase IV‐Related Peptides
Source: Food Sci Nutr. 2026 Jul 24;14(7):e72155. doi: 10.1002/fsn3.72155 (PMC13400832; doi:10.1002/fsn3.72155)
Supplement: Supplementary file 1 — Figure S1: Total ion spectrum of cheese coded 0H in LC–MS/MS. Figure S2: Total ion spectrum of cheese coded 0A in LC–MS/MS. Figure S3: Total ion spectrum of cheese coded 13H in LC–MS/MS. Figure S4: Total ion spectrum of cheese coded 13A in LC–MS/MS. Figure S5: Total ion spectrum of cheese coded 26H in LC–MS/MS. Figure S6: Total ion spectrum of cheese coded 26A in LC–MS/MS. Figure S7: Total ion spectrum of cheese coded 39H in LC–MS/MS. Figure S8: Total ion spectrum of cheese coded 39A in LC–MS/MS. Table S1: Experimental design and formulation of cheese samples enriched with soybean beverage and fermented with different starter cultures ( L. helveticus or L. acidophilus ). Table S2: Comprehensive inventory and bioactivity profiles of identified peptides derived from bovine milk and soybean proteins. Table S3: PeptideRanker scores of the identified peptides from the hybrid cheese matrices. [file FSN3-14-e72155-s001.docx]

**Supporting Information**

**Development of Functional Soybean-Enriched Cheese: Identification of Novel Angiotensin-Converting Enzyme-Inhibitory and Predicted Dipeptidyl Peptidase IV-Related Peptides**

Mehtap Er Kemal^a^, Mehmet Kemal^b^, Hasan Temiz^c^

^a^Department of Food Processing, Maçka Vocational School, Karadeniz Technical University, 61750 Trabzon, Türkiye

^b^ Department of Nutrition and Dietetics, Faculty of Health Science, 61080, Trabzon, Türkiye

^c^Department of Food Engineering, Faculty of Engineering, Ondokuz Mayıs University, 55139, Samsun, Türkiye

Corresponding author: Prof. Dr. Hasan Temiz ([hasant@omu.edu.tr](mailto:hasant@omu.edu.tr))

**Figures**

#
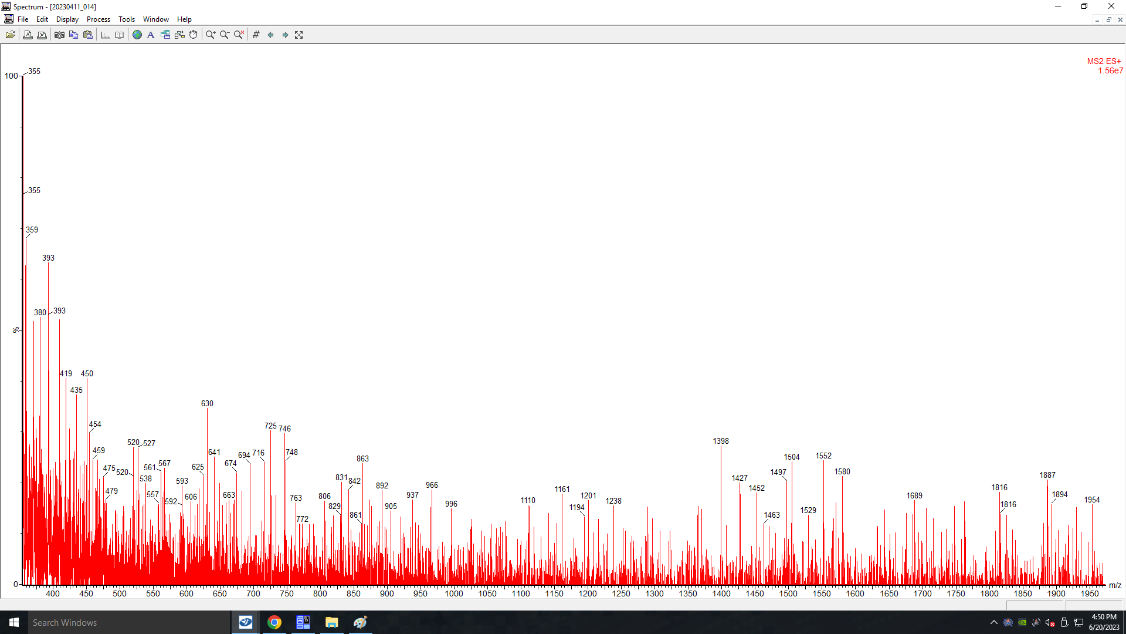


**Fig. S1** Total ion spectrum of cheese coded 0H in LC-MS/MS


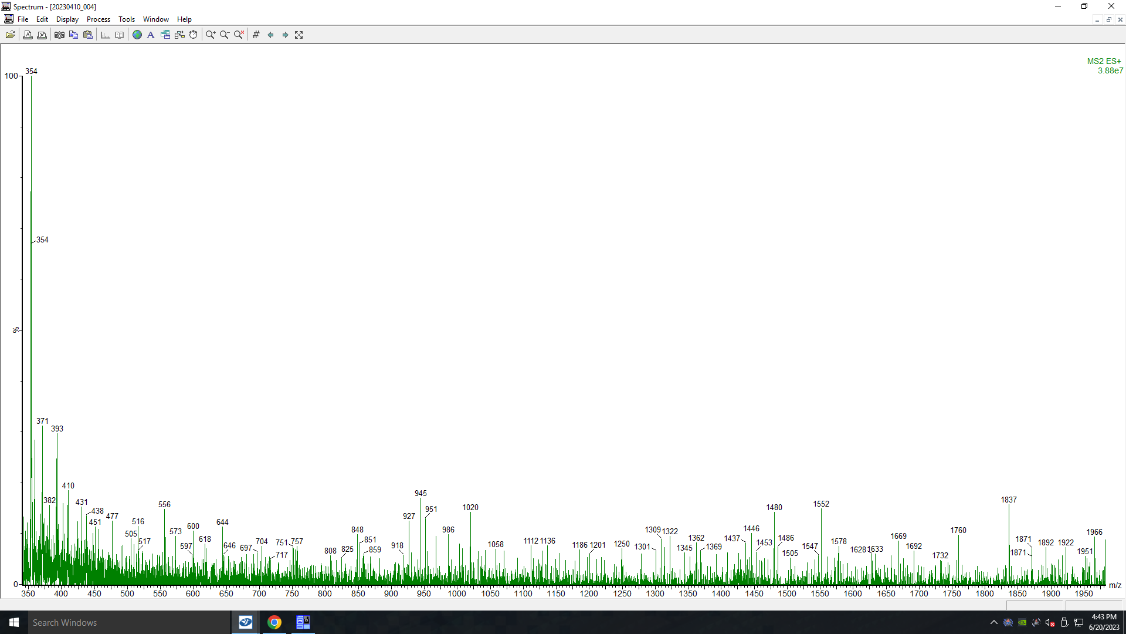


**Fig. S2** Total ion spectrum of cheese coded 0A in LC-MS/MS

#
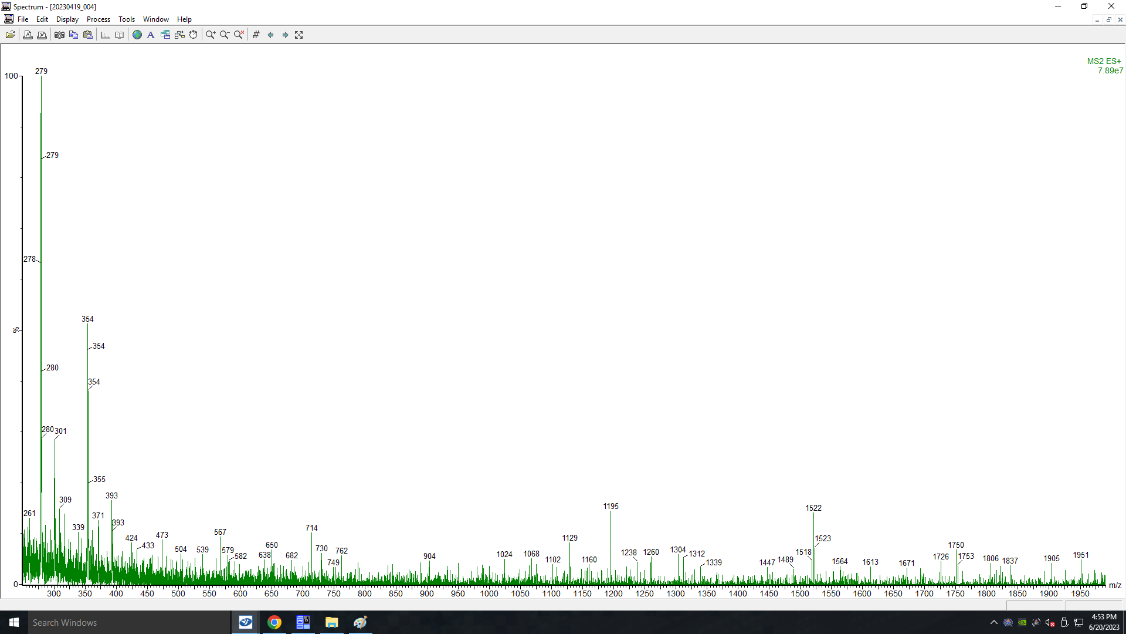


**Fig. S3** Total ion spectrum of cheese coded 13H in LC-MS/MS


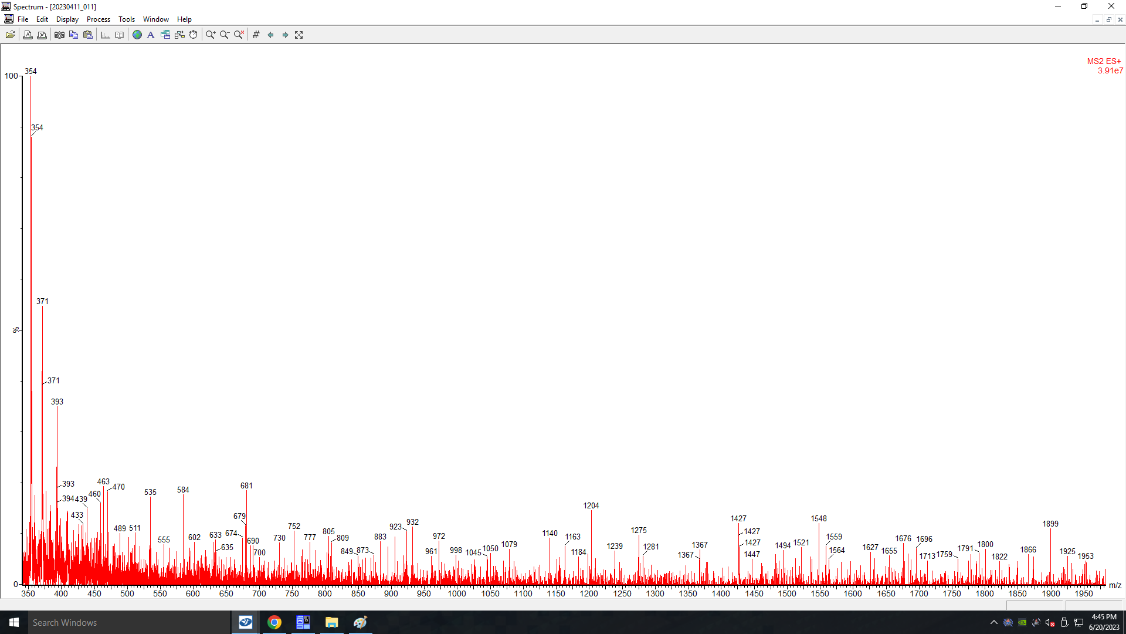


**Fig. S4** Total ion spectrum of cheese coded 13A in LC-MS/MS

#
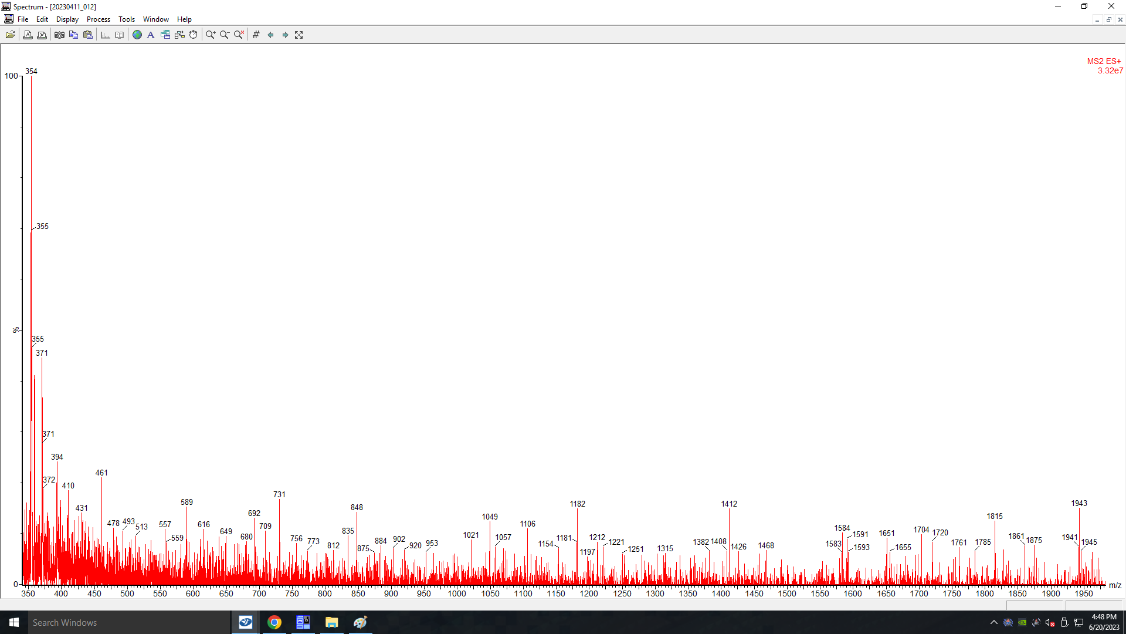


**Fig. S5** Total ion spectrum of cheese coded 26H in LC-MS/MS


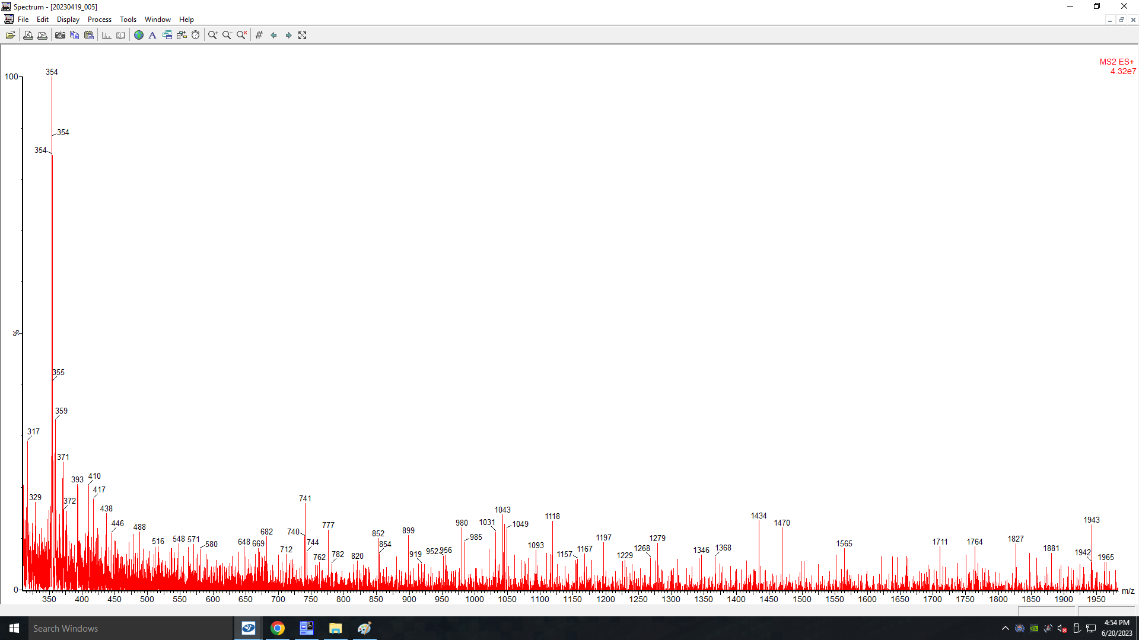


**Fig. S6** Total ion spectrum of cheese coded 26A in LC-MS/MS

#
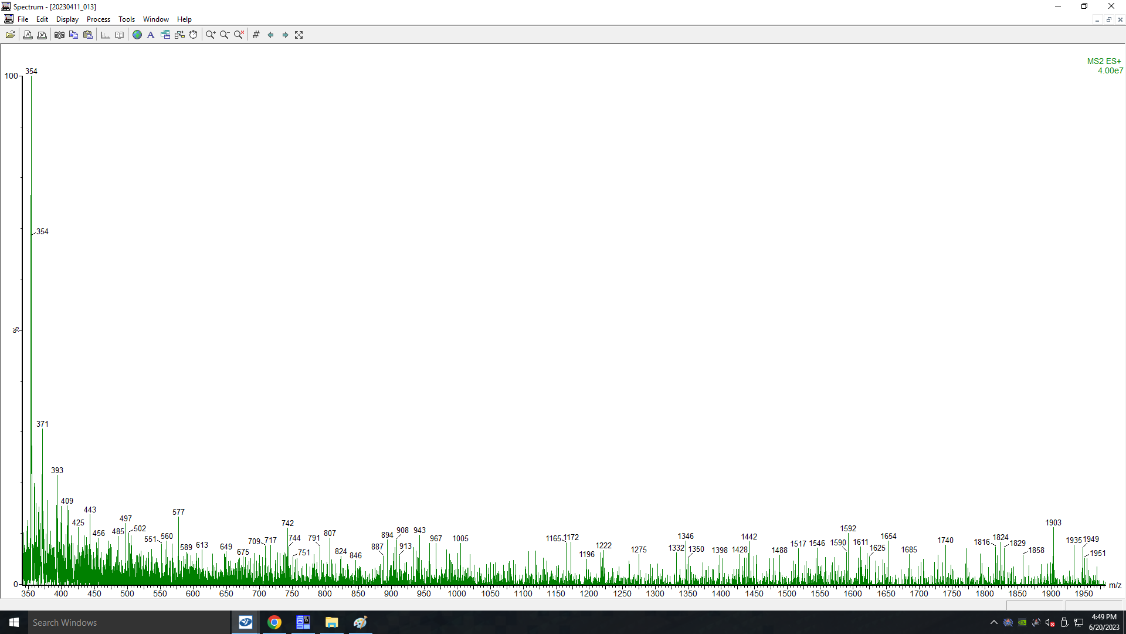


# **Fig. S7** Total ion spectrum of cheese coded 39H in LC-MS/MS

#
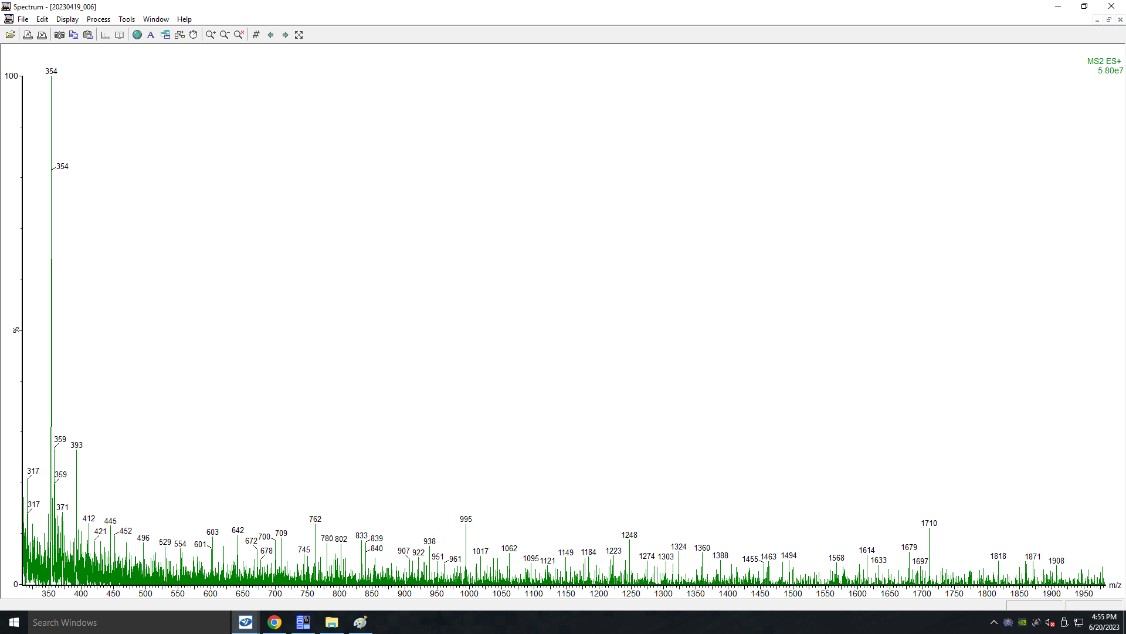


# **Fig. S8** Total ion spectrum of cheese coded 39A in LC-MS/MS

**Table S1.** Experimental design and formulation of cheese samples enriched with soybean beverage and fermented with different starter cultures (*L. helveticus* or *L. acidophilus*).

| **Code** | **Adjunct cultures** |
| --- | --- |
| H | *Lactobacillus helveticus* (1.5%)  *Lactobacillus casei* (0.5%)  *Lactobacillus cremoris* (0.5%)  *Lactobacillus lactis* (0.5%) |
| A | *Lactobacillus acidophilus* (1.5%)  *Lactobacillus casei* (0.5%)  *Lactobacillus cremoris* (0.5%)  *Lactobacillus lactis* (0.5%) |

**Table S2.** Comprehensive inventory and bioactivity profiles of identified peptides derived from bovine milk and soybean proteins

| **Protein** | **Peptide sequence** | **Molecular weight (Da)** | **Potential Bioactivity** | **Detected Samples** |
| --- | --- | --- | --- | --- |
| **α_s1_-casein** | LNENLLRFFVAPFPEVFG | 2110.46 | ACE-I | 0H45, 13H45, 26H45, 39H45, 0A45, 13A45, 26A45, 39A45 |
|  | AYFYPE | 789.86 | ACE-I | 0H45, 13H45, 26H45, 39H45, 0A45, 13A45, 26A45, 39A45 |
|  | AYFYPEL | 903.02 | ACE-I | 0H45, 13H45, 26H45, 39H45, 0A45, 13A45, 26A45, 39A45 |
|  | DAYPSGAW | 866.90 | ACE-I | 0H45, 13H45, 26H45, 39H45, 0A45, 13A45, 26A45, 39A45 |
|  | FVAPFPEVFG | 1110.29 | ACE-I | 0H45, 13H45, 26H45, 39H45, 0A45, 13A45, 26A45, 39A45 |
|  | GAW | 333.36 | ACE-I | 0H45, 13H45, 26H45, 39H45, 0A45, 13A45, 26A45, 39A45 |
|  | ENLLRFFVAPFPEVFG | 1883.20 | ACE-I | 0H45, 13H45, 26H45, 39H45, 0A45, 13A45, 26A45, 39A45 |
|  | FFVAP | 580.70 | ACE-I | 0H45, 13H45, 26H45, 39H45, 0A45, 13A45, 26A45, 39A45 |
|  | FFVAPFPEVFGK | 1385.64 | ACE-I | 0H45, 13H45, 26H45, 39H45, 0A45, 13A45, 26A45, 39A45 |
|  | LAYFYP | 773.90 | ACE-I | 0H45, 13H45, 26H45, 39H45, 0A45, 13A45, 26A45, 39A45 |
|  | NENLLRFFVAPFPEVFG | 1997.30 | ACE-I | 0H45, 13H45, 26H45, 39H45, 0A45, 13A45, 26A45, 39A45 |
|  | RYLGY | 671.77 | ACE-I | 0H45, 13H45, 26H45, 39H45, 0A45, 13A45, 26A45, 39A45 |
|  | TTMPLW | 748.92 | ACE-I | 0H45, 13H45, 26H45, 39H45, 0A45, 13A45, 26A45, 39A45 |
| **α_s2_-casein** | FPQY | 554.62 | ACE-I, Antioxidant | 0H45, 13H45, 26H45, 39H45, 0A45, 13A45, 26A45, 39A45 |
|  | ALNEINQFY | 1112.22 | ACE-I | 0H45, 13H45, 26H45, 39H45, 0A45, 13A45, 26A45, 39A45 |
|  | ALNEINQFYQK | 1368.53 | ACE-I | 0H45, 13H45, 26H45, 39H45, 0A45, 13A45, 26A45, 39A45 |
|  | AMKPW | 632.80 | ACE-I | 0H45, 13H45, 26H45, 39H45, 0A45, 13A45, 26A45, 39A45 |
|  | AMKPWIQPK | 1099.38 | ACE-I | 0H45, 13H45, 26H45, 39H45, 0A45, 13A45, 26A45, 39A45 |
|  | FALPQY | 738.86 | ACE-I | 0H45, 13H45, 26H45, 39H45, 0A45, 13A45, 26A45, 39A45 |
|  | FALPQYLK | 980.19 | ACE-I, Antioxidant | 0H45, 13H45, 26H45, 39H45, 0A45, 13A45, 26A45, 39A45 |
|  | MKP | 375.51 | ACE-I | 0H45, 13H45, 26H45, 39H45, 0A45, 13A45, 26A45, 39A45 |
|  | VPITPTL | 740.91 | DPP-IV inhibitory | 0H45, 13H45, 26H45, 39H45, 0A45, 13A45, 26A45, 39A45 |
|  | YQK | 438.50 | ACE-I | 0H45, 13H45, 26H45, 39H45, 0A45, 13A45, 26A45, 39A45 |
| **β-casein** | AQTQSLVYP | 1007.13 | ACE-I | 0H45, 13H45, 26H45, 39H45, 0A45, 13A45, 26A45, 39A45 |
|  | AVPYP | 546.64 | ACE-I | 0H45, 13H45, 26H45, 39H45, 0A45, 13A45, 26A45, 39A45 |
|  | AVPYPQR | 830.96 | ACE-I, Antioxidant, Antimicrobial | 0H45, 13H45, 26H45, 39H45, 0A45, 13A45, 26A45, 39A45 |
|  | DKIHPF | 756.87 | ACE-I, Opioid | 0H45, 13H45, 26H45, 39H45, 0A45, 13A45, 26A45, 39A45 |
|  | EMPFPK | 748.92 | ACE-I | 0H45, 13H45, 26H45, 39H45, 0A45, 13A45, 26A45, 39A45 |
|  | FAQTQSLVYP | 1154.30 | ACE-I | 0H45, 13H45, 26H45, 39H45, 0A45, 13A45, 26A45, 39A45 |
|  | IHPFAQTQSLVYP | 1501.72 | ACE-I | 0H45, 13H45, 26H45, 39H45, 0A45, 13A45, 26A45, 39A45 |
|  | KIHPFAQTQSLVYP | 1629.89 | ACE-I | 0H45, 13H45, 26H45, 39H45, 0A45, 13A45, 26A45, 39A45 |
|  | KVLILA | 656.88 | ACE-I | 0H45, 13H45, 26H45, 39H45, 0A45, 13A45, 26A45, 39A45 |
|  | RDMPIQAF | 978.16 | ACE-I | 0H45, 13H45, 26H45, 39H45, 0A45, 13A45, 26A45, 39A45 |
|  | SLPQN | 558.61 | ACE-I | 0H45, 13H45, 26H45, 39H45, 0A45, 13A45, 26A45, 39A45 |
|  | VLGP | 385.48 | ACE-I, DPP-IV inhibitory | 0H45, 13H45, 26H45, 39H45, 0A45, 13A45, 26A45, 39A45 |
| **κ-casein** | AIPPKKNQD | 1011.16 | ACE-I | 0H45, 13H45, 26H45, 39H45, 0A45, 13A45, 26A45, 39A45 |
|  | ARHPH | 617.68 | ACE-I | 0H45, 13H45, 26H45, 39H45, 0A45, 13A45, 26A45, 39A45 |
|  | DERF | 566.59 | ACE-I | 0H45, 13H45, 26H45, 39H45, 0A45, 13A45, 26A45, 39A45 |
|  | FSDKIAK | 808.95 | ACE-I | 0H45, 13H45, 26H45, 39H45, 0A45, 13A45, 26A45, 39A45 |
|  | IPI | 342.45 | DPP-IV inhibitory, Antioxidant | 0H45, 13H45, 26H45, 39H45, 0A45, 13A45, 26A45, 39A45 |
|  | IPIQY | 633.76 | DPP-IV inhibitory | 0H45, 13H45, 26H45, 39H45, 0A45, 13A45, 26A45, 39A45 |
|  | LPYPY | 652.76 | DPP-IV inhibitory | 0H45, 13H45, 26H45, 39H45, 0A45, 13A45, 26A45, 39A45 |
|  | MAIPPKK | 785.04 | ACE-I | 0H45, 13H45, 26H45, 39H45, 0A45, 13A45, 26A45, 39A45 |
|  | RYPSYG | 742.80 | ACE-I | 0H45, 13H45, 26H45, 39H45, 0A45, 13A45, 26A45, 39A45 |
|  | VLSRYP | 734.87 | ACE-I | 0H45, 13H45, 26H45, 39H45, 0A45, 13A45, 26A45, 39A45 |
| **α-lactalbumin** | LAHKAL | 652.81 | ACE-I | 0H45, 13H45, 26H45, 0A45, 13A45, 26A45 |
|  | LAHKALCSEKL | 1213.48 | DPP-IV inhibitory | 0H45, 13H45, 26H45, 0A45, 13A45, 26A45 |
|  | VGINYWLAHK | 1200.40 | ACE-I | 0H45, 13H45, 26H45, 0A45, 13A45, 26A45 |
|  | YGGVSLPEW | 1008.11 | ACE-I | 0H45, 13H45, 26H45, 0A45, 13A45, 26A45 |
|  | YGLF | 499.58 | ACE-I | 0H45, 13H45, 26H45, 0A45, 13A45, 26A45 |
|  | WLAHKAL | 839.02 | ACE-I | 0H45, 13H45, 26H45, 0A45, 13A45, 26A45 |
|  | WLAHKALCSEKLDQ | 1642.91 | DPP-IV inhibitory | 0H45, 13H45, 26H45, 0A45, 13A45, 26A45 |
| **β-lactoglobulin** | ALPM | 431.57 | ACE-I | 0H45, 13H45, 0A45, 13A45 |
|  | ALPMH | 568.71 | ACE-I | 0H45, 13H45, 0A45, 13A45 |
|  | ALPMHIR | 838.06 | ACE-I | 0H45, 13H45, 0A45, 13A45 |
|  | CMENSA | 654.74 | ACE-I | 0H45, 13H45, 0A45, 13A45 |
|  | DAQSAPLRVY | 1120.25 | ACE-I | 0H45, 13H45, 0A45, 13A45 |
|  | IIAEK | 573.71 | ACE-I | 0H45, 13H45, 0A45, 13A45 |
|  | IPAVF | 546.68 | DPP-IV inhibitory | 0H45, 13H45, 0A45, 13A45 |
|  | IPAVFKIDA | 974.18 | DPP-IV inhibitory | 0H45, 13H45, 0A45, 13A45 |
|  | IQKVAGTW | 903.06 | ACE-I | 0H45, 13H45, 0A45, 13A45 |
|  | LAMA | 405.53 | ACE-I | 0H45, 13H45, 0A45, 13A45 |
|  | LDAQSAPLR | 971.10 | ACE-I | 0H45, 13H45, 0A45, 13A45 |
|  | LKALPMH | 810.05 | ACE-I | 0H45, 13H45, 0A45, 13A45 |
|  | VAGTWY | 696.78 | ACE-I | 0H45, 13H45, 0A45, 13A45 |
|  | WYSLA | 639.72 | ACE-I | 0H45, 13H45, 0A45, 13A45 |
|  | WYSLAM | 770.92 | ACE-I | 0H45, 13H45, 0A45, 13A45 |
|  | WYSLAMA | 842.00 | ACE-I | 0H45, 13H45, 0A45, 13A45 |
| **Lactoferrin** | IPM | 360.49 | DPP-IV inhibitory | 0H45, 39H45, 0A45, 39A45 |
|  | GILRP | 555.69 | ACE-I | 0H45, 39H45, 0A45, 39A45 |
|  | LIWKL | 672.88 | ACE-I | 0H45, 39H45, 0A45, 39A45 |
|  | LNNSRAP | 771.85 | ACE-I | 0H45, 39H45, 0A45, 39A45 |
|  | LRPVAA | 626.77 | ACE-I | 0H45, 39H45, 0A45, 39A45 |
|  | PYKLRP | 773.95 | ACE-I | 0H45, 39H45, 0A45, 39A45 |
| **glycinin G2** | NRNAL | 587.65 | DPP-IV inhibitory | 26H45, 39H45, 26A45, 39A45 |
|  | DQMPRRF | 950.10 | ACE-I | 26H45, 39H45, 26A45, 39A45 |
|  | KTNDRPSIGNL | 1215.35 | DPP-IV inhibitory | 26H45, 39H45, 26A45, 39A45 |
|  | QCAGVAL | 661.80 | DPP-IV inhibitory | 26H45, 39H45, 26A45, 39A45 |
|  | REGDL | 589.62 | DPP-IV inhibitory | 26H45, 39H45, 26A45, 39A45 |
|  | ENQL | 503.53 | DPP-IV inhibitory | 26H45, 39H45, 26A45, 39A45 |
|  | AGNQEQEF | 922.92 | ACE-I | 26H45, 39H45, 26A45, 39A45 |
|  | GVNMQIVRNL | 1144.38 | DPP-IV inhibitory | 26H45, 39H45, 26A45, 39A45 |
|  | SAQYGSL | 725.77 | DPP-IV inhibitory | 26H45, 39H45, 26A45, 39A45 |
|  | AVAAKSQSDNF | 1138.22 | DPP-IV inhibitory | 26H45, 39H45, 26A45, 39A45 |
|  | PEEVIQHTF | 1100.21 | ACE-I | 26H45, 39H45, 26A45, 39A45 |
|  | NANSIIYAL | 979.12 | ACE-I | 26H45, 39H45, 26A45, 39A45 |
|  | VPPQESQ | 784.84 | DPP-IV inhibitory | 26H45, 39H45, 26A45, 39A45 |
|  | RKNAMF | 766.94 | ACE-I | 26H45, 39H45, 26A45, 39A45 |
|  | VPHYTL | 729.85 | DPP-IV inhibitory | 26H45, 39H45, 26A45, 39A45 |
|  | QEGGVL | 602.66 | DPP-IV inhibitory | 26H45, 39H45, 26A45, 39A45 |
|  | IVPQNF | 717.84 | DPP-IV inhibitory | 26H45, 39H45, 26A45, 39A45 |
|  | EYVSF | 644.70 | ACE-I | 26H45, 39H45, 26A45, 39A45 |
|  | NGRAL | 530.60 | DPP-IV inhibitory | 26H45, 39H45, 26A45, 39A45 |
|  | AGANSL | 532.57 | DPP-IV inhibitory | 26H45, 39H45, 26A45, 39A45 |
| **β-** **conglycinin subunit 1** | SHNIL | 583.66 | DPP-IV inhibitory | 26H45, 39H45, 26A45, 39A45 |
|  | SGRAIL | 616.73 | ACE-I | 26H45, 39H45, 26A45, 39A45 |
|  | NSKAIVIL | 858.06 | DPP-IV inhibitory | 26H45, 39H45, 26A45, 39A45 |
|  | QSKPNTIL | 901.05 | DPP-IV inhibitory | 26H45, 39H45, 26A45, 39A45 |
| **basic 7S globulin** | VQKGL | 544.67 | DPP-IV inhibitory | 26H45, 39H45, 26A45, 39A45 |
|  | HWANL | 640.71 | ACE-I | 26H45, 39H45, 26A45, 39A45 |
|  | QRQF | 578.64 | DPP-IV inhibitory | 26H45, 39H45, 26A45, 39A45 |
|  | MQVPVL | 686.89 | DPP-IV inhibitory | 26H45, 39H45, 26A45, 39A45 |

**Table S3.** PeptideRanker scores of the identified peptides from the hybrid cheese matrices

| **Peptide sequence** | **PeptideRanker score, > 0.50** |
| --- | --- |
| AIPPKKNQD | < 0.50 |
| ALNEINQFY | < 0.50 |
| ALNEINQFYQK | < 0.50 |
| ALPM | 0.816271 |
| ALPMH | 0.703249 |
| ALPMHIR | 0.626675 |
| AMKPW | 0.877666 |
| AMKPWIQPK | 0.624419 |
| AQTQSLVYP | < 0.50 |
| ARHPH | < 0.50 |
| AVPYP | 0.589819 |
| AVPYPQR | 0.567162 |
| AYFYPE | 0.712639 |
| AYFYPEL | 0.80788 |
| CMENSA | < 0.50 |
| DAQSAPLRVY | < 0.50 |
| DAYPSGAW | 0.738015 |
| DERF | 0.530651 |
| DKIHPF | 0.686943 |
| EMPFPK | 0.767686 |
| ENLLRFFVAPFPEVFG | 0.558848 |
| FALPQY | 0.672657 |
| FALPQYLK | 0.656968 |
| FAQTQSLVYP | < 0.50 |
| FFVAP | 0.875432 |
| FFVAPFPEVFGK | 0.864802 |
| FPQY | 0.89544 |
| FSDKIAK | < 0.50 |
| FVAPFPEVFG | 0.834541 |
| GAW | 0.95923 |
| GILRP | 0.596926 |
| IHPFAQTQSLVYP | < 0.50 |
| IIAEK | < 0.50 |
| IPAVF | 0.682152 |
| IPAVFKIDA | < 0.50 |
| IPI | < 0.50 |
| IPIQY | < 0.50 |
| IPM | 0.845096 |
| IQKVAGTW | < 0.50 |
| KIHPFAQTQSLVYP | < 0.50 |
| KVLILA | < 0.50 |
| LAHKAL | < 0.50 |
| LAHKALCSEKL | < 0.50 |
| LAMA | < 0.50 |
| LAYFYP | 0.814955 |
| LDAQSAPLR | 0.533652 |
| LIWKL | 0.743374 |
| LKALPMH | < 0.50 |
| LNENLLRFFVAPFPEVFG | 0.60544 |
| LNNSRAP | < 0.50 |
| LPYPY | 0.829798 |
| LRPVAA | < 0.50 |
| MAIPPKK | 0.506137 |
| MKP | 0.656824 |
| NENLLRFFVAPFPEVFG | 0.575855 |
| PYKLRP | 0.615702 |
| RDMPIQAF | 0.597478 |
| RYLGY | < 0.50 |
| RYPSYG | 0.58754 |
| SLPQN | < 0.50 |
| TTMPLW | 0.735459 |
| VAGTWY | < 0.50 |
| VGINYWLAHK | 0.504293 |
| VLGP | < 0.50 |
| VLSRYP | < 0.50 |
| VPITPTL | < 0.50 |
| WLAHKAL | < 0.50 |
| WLAHKALCSEKLDQ | < 0.50 |
| WYSLA | 0.687043 |
| WYSLAM | 0.828364 |
| WYSLAMA | 0.680431 |
| YGGVSLPEW | 0.509385 |
| YGLF | 0.953701 |
| YQK | < 0.50 |
| NRNAL | < 0.50 |
| DQMPRRF | 0.881856 |
| KTNDRPSIGNL | < 0.50 |
| QCAGVAL | < 0.50 |
| REGDL | < 0.50 |
| ENQL | < 0.50 |
| AGNQEQEF | < 0.50 |
| GVNMQIVRNL | < 0.50 |
| SAQYGSL | < 0.50 |
| AVAAKSQSDNF | < 0.50 |
| PEEVIQHTF | < 0.50 |
| NANSIIYAL | < 0.50 |
| VPPQESQ | < 0.50 |
| RKNAMF | 0.640517 |
| VPHYTL | < 0.50 |
| QEGGVL | < 0.50 |
| IVPQNF | < 0.50 |
| EYVSF | < 0.50 |
| NGRAL | < 0.50 |
| AGANSL | < 0.50 |
| SHNIL | < 0.50 |
| SGRAIL | 0.511694 |
| NSKAIVIL | < 0.50 |
| QSKPNTIL | < 0.50 |
| VQKGL | < 0.50 |
| HWANL | 0.686152 |
| QRQF | 0.683703 |
| MQVPVL | < 0.50 |
